# Prognostic Value of the Systemic Immune Inflammation Index after Thoracic Endovascular Aortic Repair in Patients with Type B Aortic Dissection

**DOI:** 10.1155/2023/2126882

**Published:** 2023-02-17

**Authors:** Yufei Zhao, Junhao Jiang, Ye Yuan, Xiaolong Shu, Enci Wang, Weiguo Fu, Lixin Wang

**Affiliations:** ^1^Department of Vascular Surgery, Zhongshan Hospital, Fudan University, Shanghai 200032, China; ^2^Vascular Surgery Institute of Fudan University, Shanghai 200032, China; ^3^National Clinical Research Center for Interventional Medicine, Shanghai 200032, China; ^4^Department of Vascular Surgery, Xiamen Branch, Zhongshan Hospital, Fudan University, Xiamen 361015, China

## Abstract

The study aimed at investigating the association between postoperative inflammatory scores and aorta-related adverse events (AAEs) after thoracic endovascular aortic repair (TEVAR) for patients with type B aortic dissection (TBAD). This single-centre, retrospective cohort included all patients who underwent TEVAR for TBAD between November 2016 and November 2020 at a university hospital. The risk factors for AAEs were analyzed by Cox proportional hazards model regression. Prediction accuracy was assessed using the area under the receiver operating characteristic curves. This study included 186 patients with a mean age of 58.5 years and a median follow-up period of 26 months. A total of 68 patients developed AAEs. Age and postoperative systemic immune inflammation index (SII) (>2893) were associated with post-TEVAR AAEs (hazard ratio (HR) 1.03, *p* = 0.003; HR 1.88, *p* = 0.043, respectively). Increased postoperative SII and age are independent risk factors for AAE post-TEVAR in patients with TBAD.

## 1. Introduction

Aortic dissection (AD) has long been managed by endovascular repair and has drawn significant research interest. The recent European Society of Cardiology (ESC) guidelines assigned thoracic endovascular aortic repair (TEVAR) as a class 1C recommendation for the treatment of complicated type B AD (TBAD) [1]. Approximately one-third of acute TBADs are reportedly complicated with malperfusion syndrome (either peripheral or visceral ischemia or hemodynamic instability) resulting in a high mortality if untreated, and furthermore, 20%-50% of the patients who survive the acute phase may develop a descending thoracic aorta aneurysm within 5 years [2–7].

Amounting evidence indicates that a surged perioperative systemic inflammatory status significantly determines the postinterventional outcomes after TEVAR for TBAD [8]. However, inflammatory markers are yet to be incorporated in any risk stratification model of AD [9–11]. Therefore, a comprehensive analysis of inflammatory scores is composed of postoperative inflammatory markers demanding a sufficiently size cohort of patients treated with TEVAR for TBAD.

We speculated that postoperative inflammatory scores would be valid indicators of the long-term prognosis of AD. This study is aimed at elucidating the potential association between the profile of postoperative inflammatory scores and post-TEVAR outcomes of TBAD, namely, mortality, endoleak, retrograde type A AD (RTAD), distal stent-induced new entry (dSINE), thoracic or distal aortic expansion, and branch artery expansion or stenosis during follow-up.

## 2. Methods

### 2.1. Study Participants

The baseline clinical data were retrospectively collected, in patients with type B aortic dissection (TBAD) who underwent thoracic endovascular aortic repair (TEVAR) at Zhongshan Hospital of Fudan University between November 2016 and November 2020. The patients were diagnosed as TBAD by computed tomography angiography (CTA) and underwent TEVAR after giving the written informed consent. The exclusion criteria included the following: (1) previous open surgery involving ascending aorta; (2) conditions that may affect the peripheral blood cells count, such as acute infections along with anti-inflammatory therapy within last three months, as well as malignant tumors, hemopoietic system disorders, or autoimmune diseases; (3) postoperative laboratory data were unavailable for review at the time of data collection; and (4) the patients who were lost to follow-up after discharge for more than 1 year.

### 2.2. Exposure Definition

The neutrophil-to-lymphocyte ratio (NLR) referred to the number of neutrophils divided by the number of lymphocytes. The monocyte-to-lymphocyte ratio (MLR) specified the number of monocytes divided by the number of lymphocytes. The platelet-to-lymphocyte ratio (PLR) was defined as the number of platelets divided by the number of lymphocytes. The systemic immune-inflammation index (SII) implied the platelet count multiplied by the NLR [12]. The systemic inflammation response index (SIRI) signaled the monocyte count multiplied by the NLR [13]. The first postoperative venous blood specimens were collected, which were mostly drawn within 24 h after surgery.

### 2.3. Outcome Measures

The occurrence of aorta-related adverse events (AAEs) during the post-TEVAR follow-up was identified by CTA, which included all types of endoleaks, RTAD, dSINE, branch artery stenosis, distal aortic expansion, aortic rupture, and death. Data were reviewed by two independent authors on a single-blind basis.

### 2.4. Potential Confounders

The potential confounding variables before enrollment or at cohort entry were evaluated. The comorbidities before enrollment include hypertension, smoking and drinking histories, diabetes mellitus, coronary artery disease including myocardial infarction, heart failure, ischemic or hemorrhagic stroke, chronic kidney disease, and peripheral artery disease. The medication history of antiplatelets, anticoagulants, antihypertensive drugs, and lipid-lowering drugs in the year before cohort entry was also considered. Stent-graft-related variables including the intervention phase (acute, subacute, or chronic), the location of primary tear (Z3 or Z4), the length of proximal landing zone (the distance between primary tear and the left subclavian artery), and the adjunctive stent-graft measures (in situ fenestration or chimney, etc.) were documented between diagnosis and cohort entry.

### 2.5. Statistical Analysis

We calculated that a sample of 186 patients (68 in the AAE group and 118 in the non-AAE group) would provide the study with over 90% power to detect a difference in the AAE incidence between the AAE and non-AAE groups (assuming AAE incidence as 20% in the AAE group and 10% in the non-AAE group) at a two-sided significance level of 0.05. Continuous variables are summarized as medians and interquartile ranges (IQRs) or means and standard deviations (SDs). Categorical variables are presented as numbers and percentages. Comparisons between groups were made using Pearson's chi-square test or Fisher's exact test for categorical variables while Student's *t*-test or the Mann–Whitney *U* test for continuous variables. The Kolmogorov–Smirnov test was used to test the normality of variables. The optimal cut-off values of the quantitative variables were determined by receiver operating characteristic (ROC) curve. The Kaplan–Meier curves with the log-rank test were used to perform survival analysis and assess the differences in the time-to-event endpoints. To identify independent risk factors, statistically significant risk factors (*p* < 0.20) on univariable analysis were further incorporated in the multivariable Cox proportional hazards model regression analysis. Population restriction was conducted to assess the robustness of our findings, and missing data were handled with multiple imputation (because of study dropouts and incomplete patient interviews) (Supplementary Table 1 and 2). Data analysis and visualization were performed using R (R Foundation for Statistical Computing, Vienna, Austria) and GraphPad Prism 8.0 (GraphPad Software, San Diego, CA, USA).

## 3. Results

### 3.1. Baseline Characteristics

**Figure 1:**
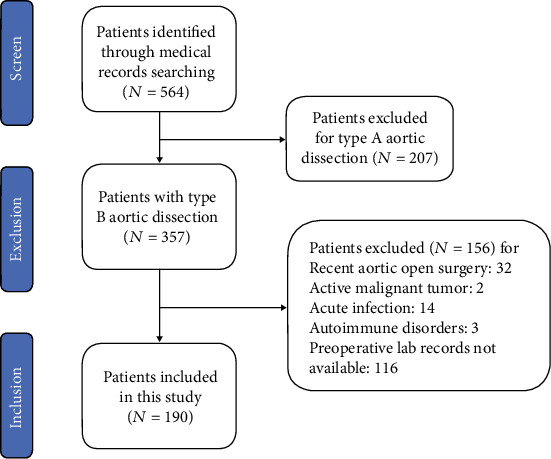
Flow diagram according to the Preferred Reporting Items.

The inclusion and exclusion of patients are shown in flowchart (Figure 1). A total of 186 patients with type B AD were included, with a mean age of 58.5 ± 10.3 years and a median (IQR) follow-up period of 26.2 (12.1-39.3) months. Among them, 68 patients developed aorta-related adverse events (AAEs) after thoracic endovascular aortic repair (TEVAR), including twenty endoleaks (Ia, Ib, and III), one RTAD, 6 distal stent-induced new entries, 5 visceral artery stenoses, 4 left subclavian artery stenoses or expansions, 1 thoracic aortic expansion, 29 distal abdominal aortic expansions leading to distal aneurysm, and 1 death due to aortic rupture during hospitalization. The six-month, one-year, and three-year incidences of AAEs during the post-TEVAR follow-up were 0.05% (*n* = 10), 13.68% (*n* = 26), and 28.42% (*n* = 54), respectively.

Table 1 shows the baseline characteristics of patients in the AAE and non-AAE groups. Patients who developed AAEs following TEVAR had a mean age of 66.0 ± 11.4 years, which was higher than that of patients in the non-AAE group (*p* = 0.001). No statistically significant differences were observed between male and female patients (*p* = 0.407). The prevalence of hypertension, which is the most common risk factor for TBAD (75.3%), differed between the AAE and non-AAE groups (*p* = 0.034), whereas the history of coronary artery disease, chronic kidney disease, diabetes mellitus, and stroke, the history of smoking and drinking, and the use of antiplatelet, anticoagulant drugs, or lipid-lowering drugs (mostly statins) showed no difference between the AAE and non-AAE groups (*p* > 0.05). Likewise, the intervention timepoint, the length of proximal landing zone, the location of primary tear, and the adjunctive stent-graft measures exhibited no differences between the AAE and non-AAE groups (*p* > 0.05).

Compared with the non-AAE group, the patients with AAEs had lower lymphocyte counts (*p* = 0.045) and decreased platelets (*p* = 0.019). The triglyceride levels in the patients without AAEs were higher than those in the AAE group (*p* = 0.043). Nonetheless, no significant differences were observed between the AAE and non-AAE groups in terms of leukocytes, neutrophils, monocytes, hemoglobin, D-dimer, fibrinogen, albumin, serum creatinine, cholesterol, low-density lipoprotein, or high-density lipoprotein levels.

The postoperative NLR and SII were different between the AAE and non-AAE groups (*p* = 0.023 and *p* = 0.016, respectively). The MLR, PLR, and SIRI did not differ with statistical significance between both groups (*p* = 0.300, 0.200, and 0.110, respectively).

### 3.2. Postoperative Inflammatory Biomarkers and AAEs

**Figure 2:**
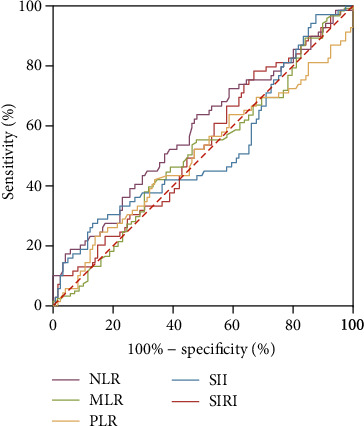
A receiver operating characteristic curve to explore the value of postoperative inflammatory biomarkers NLR, MLR, PLR, SII, and SIRI to identify AAEs after thoracic endovascular aortic repair (TEVAR).

The association between postoperative inflammatory scores and AAEs after TEVAR was studied by receiver operating characteristic (ROC) curve, and thereby, optimal cut-off values of five inflammatory scores can be determined (Figure 2). The areas under the ROC curves (AUC) for NLR, MLR, PLR, SII, and SIRI were 0.584, 0.509, 0.502, 0.520, and 0.533, respectively, indicating moderate performances for the prediction of post-TEVAR AAEs. The sensitivity and specificity of each inflammatory biomarker for predicting AAEs are shown in Table 2.

Univariable logistic regression revealed that age and elevated NLR were potential risk factors for post-TEVAR AAEs (OR 1.051, *p* = 0.001 and OR 1.080, *p* = 0.009, respectively) (Table 3). Similarly, multivariable logistic regression analysis further verified that SII and age were independent risk factors following TEVAR for AAEs (OR 1.05, *p* = 0.001; OR 1.40, *p* = 0.42, respectively). Besides, the SII was indicated in the multivariable logistic regression as a potential risk factor with a *p* value of 0.04 and OR of 2.48 (Table 4).

Moreover, the age, NLR, and SII were closely associated with AAEs in univariable Cox proportional hazard regression analysis (HR 1.03, *p* = 0.003; HR 1.77, *p* = 0.024; and HR 2.12, *p* = 0.006, respectively). The multivariable Cox regression analysis ultimately confirmed that SII and age were independent risk factors for AAEs (HR 1.03, *p* = 0.003 and HR 1.88, *p* = 0.043, respectively) (Table 5).

**Figure 3:**
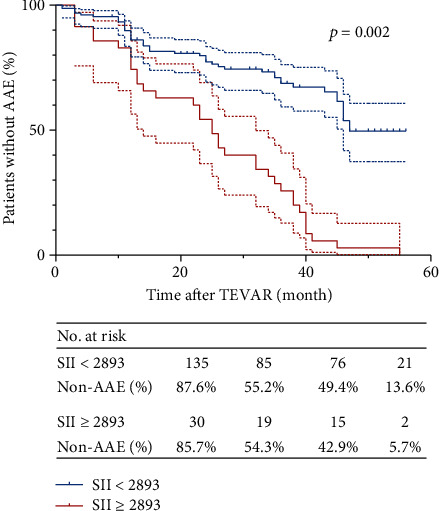
The Kaplan–Meier AAE-free survival (AFS) curves after thoracic endovascular aortic repair (TEVAR) with the SII of >2893 versus <2893. Dashed lines indicate the upper and lower limits for 95% confidence interval (CI).

Figure 3 shows the Kaplan–Meier AAE-free survival (AFS) curves for SII. The median follow-up period for the postoperative SII ≥ 2893 group was 27 months, and the median follow-up period for the postoperative SII < 2893 group was 32 months. The median AAE-free survival for the postoperative SII ≥ 2893 group was 24.7 ± 13.7 months, while the median AAE-free survival for the postoperative SII < 2893 group was 27.9 ± 16.2 months. Patients with a postoperative SII ≥ 2893 had considerably lower ten-month, twenty-month, and forty-month AFS than those with SII < 2893 in survival analysis (*p* < .001).

**Figure 4:**
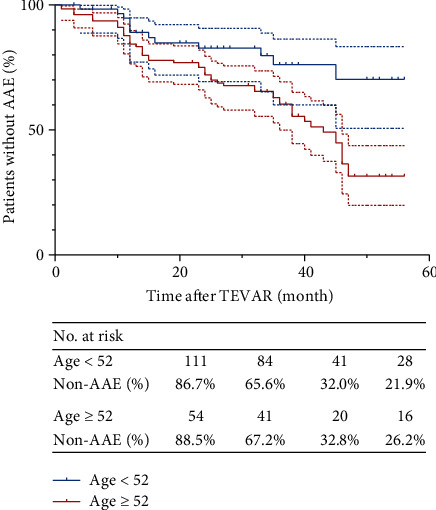
The Kaplan–Meier AAE-free survival (AFS) curves after thoracic endovascular aortic repair (TEVAR) with the age of >52 versus <52. Dashed lines indicate the upper and lower limits for 95% confidence interval (CI).

Figure 4 shows the Kaplan–Meier AFS curves for age. The median follow-up period for patients aged ≥52 group was 31 months, and the median follow-up period for those aged <52 group was 32 months. The median AFS of patients aged ≥52 was 26.7 ± 15.6 months, and the median AFS of those aged <52 was 28.7 ± 16.1 months. From the survival analysis, significantly lower AFS at ten months, twenty months, and forty months was revealed in patients with postoperative SII ≥ 2893 than in those with a SII < 2893 (*p* < .005).

In addition, the association between postoperative SII and reintervention after TEVAR was studied. The Kaplan–Meier survival curves showed that several patients were free from reintervention (Supplementary Figure 1). Survival analysis revealed no statistical differences of reintervention in patients with postoperative SII ≥ 2893 compared with patients with SII < 2893 (log-rank test, *p* < 0.001).

## 4. Discussion

Previous studies have corroborated the prognostic significance of platelets and NLR in patients with AD [14–17]. In this cohort of patients with TBAD, a new inflammation-immune-based score (SII) was developed based on neutrophil, lymphocyte, and platelet counts [12], and it shows that postoperative SII is an independent risk factor for AAEs following TEVAR, and the risk of patients with SII ≥ 2893 of developing adverse events was increased by a factor of 1.8 compared to patients with SII below 2893 (Graphical Abstract/Supplementary Figure 2).

In our study, a postoperative SII greater than 2893 was largely due to higher platelet levels, although the monocyte counts increased and lymphocyte counts declined. The MLR was not statistically significant after multivariable Cox regression, indicating a more important role for platelets in the risk stratification of AD prognosis.

To reveal the nature of the relevance between a high SII levels and a poor outcome is beyond the scope of this observational study. However, in view of the function of the three kinds of cells, a possible explanation may lie in the long-term aortic remodeling of a partial thrombosis within the false lumen. Platelets actively participate in thrombus formation, while thrombosis in the false lumen initiated by TEVAR may eventually result in aortic remodeling and platelet consumption. Therefore, a high SII may indicate partial thrombosis of the false lumen or a patent false lumen after TEVAR, which is considered as a significant independent predictor of poor long-term outcome in AD patients [18–21], and account for the exacerbation of aortic remodeling.

The link between platelets and postoperative complications of AD has been previously addressed. Initially, platelet dysfunction [21] and decreased platelet counts [15, 16] were observed in patients with TAAD. Low platelet counts at admission have been reported as a strong predictor of increased in-hospital mortality in TAAD patients with or without surgical intervention [15]. In a large retrospective cohort study of 744 patients with TAAD, individual biomarkers of platelets, neutrophils, and lymphocytes failed to predict 30-day mortality alone, while in combination, they provided the strongest predictive value of 30-day mortality [17]. In contrast to our findings, however, low platelets were associated with a poor prognosis in several previous studies, which may be attributable to the following reasons. First, the main outcome of previous studies was 30-day mortality, which is a short-term endpoint that provides a limited window for assessing the incidence of adverse events, considering that numerous long-term complications may occur after surgery for TAAD. Second, the cohort included patients with or without surgical intervention. In routine clinical practice, patients eligible for surgical treatment often undergo emergency life-saving surgery, while there are patients who cannot undergo open thoracotomy and aortic repair due to severe and irreversible organ damage or poor general condition. Thus, those patients without surgery are prone to have unstable vital signs, shock, and disseminated intravascular coagulation, contributing to coagulation disturbance, which in turn reduces platelet counts.

One fundamental but underappreciated concept that “not statistically different” does not necessarily mean “the same” [22] reminds us not to ignore the rest of the seemingly meaningless biomarkers, particularly neutrophils and lymphocytes, as part of the SII in the regression model. Recent findings have extended the specific functions of platelets in the context of immune-driven mechanisms, such as platelet-leukocyte interactions, which appear to be a previously unrecognized bridge of two essential pathophysiological processes: thrombosis and inflammation [17, 23]. Neutrophil extracellular traps (NETs), composed of extracellular DNA fibers, histones, and neutrophil antimicrobial proteins, have been proven beneficial for platelet aggregation [24], and vice versa; platelets have been shown to directly promote the production of NETs [25–27], strongly suggesting a positive feedback loop between platelets and NETs. Moreover, activated platelets, platelet-leukocyte aggregates, and neutrophil infiltration in the culprit lesions of aorta have been identified in human tissue samples, representing an ideal proof to explain the inseparability of thrombotic and inflammatory processes. In addition, inflammatory cytokines, released from recruited or residing inflammatory cells in locally dissected and weakened aorta, indicated a strengthened systemic inflammatory status and impaired immune response, as evidenced by the varying degrees of inflammatory biomarkers in the peripheral blood. The NLR is a readily available inflammatory biomarker that independently predicts cardiovascular risk and all-cause mortality in patients with atherosclerosis [28] and early adverse events in AD patients undergoing TEVAR [14]. Given its statistical significance in this and several retrospective cohorts, the predictive value of NLR in both short- and long-term prognoses of TBAD should be meticulously scrutinized and validated in future prospective cohort studies.

There are some limitations concerning this study. First, we incorporated inflammatory biomarkers from the first blood test following TEVAR, rather than the average levels of inflammatory biomarkers during the entire hospitalization episode. This may introduce a bias to a complete understanding of the varying range of systemic inflammatory status in patients. Second, the AUCs for the five inflammatory biomarkers were over 50%, indicating weak performance for the prediction of AAEs. Although statistically significant differences were observed, the association between inflammatory biomarkers and long-term prognosis of patients with TBAD warrants further evaluation in a larger prospective cohort.

## 5. Conclusion

In this study, we identified a novel prognostic score—SII—as an independent risk factor for aorta-related adverse events (AAEs) following TEVAR in patients with TBAD. With improved understanding of risk factors and the possible mechanisms behind them (such as platelet-leukocyte interaction), AAEs after TEVAR may in the future become predictable and even preventable.

## Figures and Tables

**Table 1:** Baseline characteristics.

	General population (*n* = 186)	AAE group (*n* = 68)	Non-AAE group (*n* = 118)	*p* value
*Demographics*				
Age	58.5 ± 10.3	66.0 ± 11.4	54.6 ± 12.7	0.001^†^
Gender				0.303^‡^
Male	149	52 (76.8)	97 (81.8)	
Female	37	16 (23.2)	21 (18.2)	
*Histories and comorbidities*				
Hypertension	143 (75.3)	58 (84.1)	85 (70.2)	0.034^†^
SBP	79 (41.6)	30 (43.5)	49 (40.5)	0.688^†^
DBP	80 (42.3)	35 (50.7)	45 (37.2)	0.069^†^
Smoking	54 (28.4)	21 (30.4)	33 (27.3)	0.642^‡^
Alcohol	22 (11.7)	5 (7.2)	17 (14.0)	0.159^‡^
Diabetes	16 (8.9)	4 (5.8)	12 (10.6)	0.250^‡^
History of CAD	9 (4.7)	1 (1.4)	8 (6.6)	0.107^‡^
History of AMI	1 (0.5)	0 (0)	1 (0.8)	0.341^‡^
History of stroke or TIA	6 (3.2)	1 (1.4)	5 (4.1)	0.558^‡^
History of CKD	6 (3.2)	2 (2.9)	4 (3.3)	0.877^‡^
*Medication on admission*				
Antiplatelet	8 (4.2)	2 (2.9)	6 (5.0)	0.497^‡^
Anticoagulant	0 (0)	0 (0)	0 (0)	—
Statin	5 (2.6)	1 (1.4)	4 (3.3)	0.766^‡^
*Intervention*				
Intervention phase	163 (87.4)	60 (36.7)	103 (36.7)	0.923^‡^
Acute	125 (76.7)	46 (75.4)	79 (76.7)	
Subacute	26 (15.7)	9 (15.0)	17 (16.5)	
Chronic	12 (7.2)	5 (8.3)	7 (6.7)	
The location of primary tear	131 (68.9)	46 (35.1)	85 (64.9)	0.719^‡^
Z3	122 (93.1)	42 (91.3)	80 (94.1)	
Z4	9 (6.9)	4 (2.2)	5 (5.9)	
PLZ (cm)	1.76 ± 0.42	1.77 ± 0.40	1.75 ± 0.42	0.841^†^
Adjunctive procedure	9 (4.7)	4 (5.8)	5 (4.1)	0.726^‡^
*Postoperative hematological parameters*				
Leucocyte (10 × 10^9^/L)	90 (48.4)	35 (51.5)	55 (46.6)	0.498^†^
Neutrophil (6.3 × 10^9^/L)	135 (72.6)	51 (75.0)	84 (71.2)	0.612^†^
Monocyte (0.6 × 10^9^/L)	144 (77.4)	50 (73.5)	94 (79.7)	0.307^†^
Lymphocyte (1.1 × 10^9^/L)	80 (43.0)	36 (52.9)	44 (37.3)	0.038^†^
Hemoglobin (<130 g/L)	101 (54.3)	39 (57.4)	62 (52.5)	0.120^‡^
Platelet (<125)	22 (11.8)	13 (19.1)	9 (7.6)	0.019^‡^
D-dimer	89 (97.8)	35 (50.7)	54 (44.6)	0.418^†^
Fibrinogen (>400)	111 (60.0)	40 (58.0)	71 (58.7)	0.924^‡^
Albumin (<35)	41 (22.2)	19 (27.5)	22 (18.2)	0.132^‡^
Serum creatinine	43 (22.6)	16 (23.2)	27 (22.3)	0.890^‡^
GFR (<100)	124 (65.3)	45 (65.2)	79 (65.3)	0.992^‡^
Cholesterol	17 (8.9)	7 (10.1)	10 (8.3)	0.662^‡^
Triglycerides	30 (15.8)	6 (8.7)	24 (19.8)	0.043^‡^
LDL	13 (6.8)	4 (5.8)	9 (7.4)	0.667^‡^
HDL	40 (21.1)	9 (13.0)	31 (25.6)	0.063^‡^
NLR (>6.703)	100 (53.7)	44 (64.7)	56 (47.5)	0.023^‡^
MLR (>0.591)	117 (62.9)	46 (67.6)	71 (60.2)	0.300^‡^
PLR (<113.8)	32 (36.8)	15 (22.1)	17 (14.4)	0.200^‡^
SII (>2893)	35 (18.4)	19 (27.9)	16 (13.6)	0.016^‡^
SIRI (>3.776)	132 (71.1)	53 (77.9)	79 (66.9)	0.110^‡^

SBP = systolic blood pressure; DBP = diastolic blood pressure; CAD = coronary artery disease; AMI = acute myocardial ischemia; CVD = cerebrovascular disease; TIA = transient ischemic attack; CKD = chronic kidney disease; eGFR = estimated glomerular filtration rate; LDL = low-density lipoprotein; HDL = high-density lipoprotein; WBC = white blood cell; NLR = neutrophil-to-lymphocyte ratio; MLR = monocyte-to-lymphocyte ratio; PLR = platelet-to-lymphocyte ratio; SII = the ratio of platelet count multiply neutrophil count to lymphocyte count; SIRI = the ratio of monocyte count multiply neutrophil count to lymphocyte count. ^†^Student's *t*-test or Mann–Whitney test. ^‡^Pearson's chi-square test or Fisher's exact test.

**Table 2:** Receiver operating characteristic curve analysis.

	Cut-off	AUC	Sensitivity	Specificity	PPV	NPV	Accuracy	*f*1 score
Age	>51.50	0.5829	0.45	0.82	0.82	0.44	0.58	0.58
NLR	>6.703	0.5841	0.64	0.73	0.47	0.84	0.71	0.54
MLR	<0.591	0.5090	0.20	0.39	0.35	0.23	0.27	0.26
PLR	<113.8	0.5020	0.67	0.65	0.08	0.97	0.65	0.16
SII	>2893	0.5204	0.38	0.63	0.07	0.93	0.62	0.12
SIRI	>3.776	0.5336	0.63	0.81	0.70	0.76	0.74	0.67

NLR = neutrophil-to-lymphocyte ratio; MLR = monocyte-to-lymphocyte ratio; PLR = platelet-to-lymphocyte ratio; SII = systemic immune inflammation index; SIRI = systemic inflammatory response index; OR = odds ratio; CI = confidence interval; AUC = the area under the receiver operating characteristic curve; PPV = positive prediction value; NPV = negative prediction value.

**Table 3:** Univariable logistic regression.

	OR (95% CI)	*p* value
*Demographics*		
Age	1.051 (1.017-1.072)	0.001
Gender	0.736 (0.356-1.520)	0.408
*Risk factors and comorbidities*		
SBP	0.998 (0.987-1.009)	0.721
DBP	0.997 (0.988-1.006)	0.564
Smoking	1.167 (0.609-2.236)	0.642
Alcohol	0.473 (0.167-1.346)	0.161
Diabetes		
History of CAD	0.206 (0.025-1.682)	0.140
History of AMI	—	—
History of stroke	0.341 (0.039-2.982)	0.331
History of CKD	0.873 (0.156-4.894)	0.877
*Medication on admission*		
Statin	0.426 (0.047-3.894)	0.450
Antiplatelet	0.572 (0.112-2.915)	0.502
Anticoagulant	—	—
*Preoperative hematological parameters*		
Leucocyte	1.030 (0.939-1.131)	0.529
Neutrophil	1.070 (0.983-1.164)	0.119
Monocyte	0.584 (0.269-1.269)	0.175
Lymphocyte	0.794 (0.463-1.364)	0.404
Hemoglobin	0.994 (0.978-1.009)	0.420
Platelet	0.998 (0.994-1.001)	0.155
D-dimer	1.060 (0.988-1.137)	0.105
Fibrinogen	0.999 (0.997-1.000)	0.127
Albumin	0.898 (0.933-0.969)	0.505
Serum creatinine	1.000 (0.998-1.003)	0.872
Cholesterol	0.898 (0.774-1.042)	0.155
Triglycerides	0.735 (0.504-1.071)	0.109
LDL	0.815 (0.642-1.036)	0.095
HDL	0.861 (0.550-1.346)	0.511
NLR (>6.703)	1.080 (1.019-1.145)	0.009
MLR (<0.591)	1.161 (0.610-2.232)	0.656
PLR (<113.8)	1.001 (0.987-1.002)	0.163
SII (>2893)	1.000 (1.000-1.000)	0.122
SIRI (>3.776)	1.024 (0.965-1.074)	0.331

SBP = systolic blood pressure; DBP = diastolic blood pressure; CAD = coronary artery disease; AMI = acute myocardial ischemia; CVD = cerebrovascular disease; CKD = chronic kidney disease; eGFR = estimated glomerular filtration rate; LDL = low-density lipoprotein; HDL = high-density lipoprotein; WBC = white blood cell; NLR = neutrophil-to-lymphocyte ratio; MLR = monocyte-to-lymphocyte ratio; PLR = platelet-to-lymphocyte ratio; SII = systemic immune inflammation index; SIRI = systemic inflammatory response index; OR = odds ratio; CI = confidence interval.

**Table 4:** Multivariable logistic regression.

	OR (95% CI)	*p* value
Age	1.05 (1.02-1.08)	0.001
NLR	1.40(0.61-3.19)	0.42
MLR	0.73 (0.25-2.05)	0.54
PLR	1.87 (0.77-4.51)	0.16
SII	2.48 (1.02-6.14)	0.04
SIRI	2.33 (0.75-7.32)	0.14

NLR = neutrophil-to-lymphocyte ratio; MLR = monocyte-to-lymphocyte ratio; PLR = platelet-to-lymphocyte ratio; SII = systemic immune inflammation index; SIRI = systemic inflammatory response index; OR = odds ratio; CI = confidence interval.

**Table 5:** Univariable and multivariable Cox regression.

	Univariable Cox regression		Multivariable Cox regression	
	HR (95% CI)	*p* value	HR (95% CI)	*p* value
Age (>51.50)	1.03 (1.00-1.05)	0.003	1.03 (1.01-1.04)	0.003
NLR (>6.703)	1.77 (1.08-2.92)	0.024	—	0.31
MLR (>0.591)	—	0.734	—	—
PLR (<113.8)	—	0.422	—	—
SII (>2893)	2.12 (1.24-3.64)	0.006	1.88 (1.02-3.48)	0.043
SIRI (>3.776)	—	0.286	—	—

NLR = neutrophil-to-lymphocyte ratio; MLR = monocyte-to-lymphocyte ratio; PLR = platelet-to-lymphocyte ratio; SII = systemic immune inflammation index; SIRI = systemic inflammatory response.

## Data Availability

The data are not publicly available due to privacy or ethical restrictions. The data that support the findings of this study are available from the corresponding authors upon reasonable request.

## Glossary

AD: Aortic dissection
AUC: Area under the ROC curve
AAEs: Aorta-related adverse events
dSINE: Distal stent-induced new entry
IRAD: International Registry of Acute Aortic Dissection
NLR: Neutrophil-to-lymphocyte ratio
MLR: Monocyte-to-lymphocyte ratio
PLR: Platelet-to-lymphocyte ratio
ROC: Receiver operating characteristic curve
RTAD: Retrograde type A aortic dissection
SII: Systemic immune inflammation index
SIRI: Systemic inflammatory response index
TAAD: Type A aortic dissection
TBAD: Type B aortic dissection
TEVAR: Thoracic endovascular aortic repair.

## Supplementary Materials

Supplementary Materials Supplementary Figure 1: the Kaplan–Meier reintervention-free survival curves after thoracic endovascular aortic repair (TEVAR) with the SII of >2893 versus <2893. Dashed lines indicate the upper and lower limits for 95% confidence interval (CI). Graphical Abstract/Supplementary Figure 2: although perioperative inflammation has a role in predicting postoperative outcomes after thoracic endovascular aortic repair (TEVAR) for type B aortic dissection, yet inflammatory biomarkers have not been incorporated in any risk stratification model. Therefore, a further study of postoperative inflammatory biomarkers is demanded. This study reveals that elevated postoperative systemic immune inflammation index (SII) and age are independent risk factors for aorta-related adverse events after TEVAR in type B aortic dissection. It is indicated that SII, an easily measured biomarker in clinical practice, has a certain boundary value, beyond which the risk of aorta-related adverse events approximately doubled. Supplementary Table 1: the study population was restricted to patients with acute aortic dissection to minimize the potential difference between acute and chronic dissection. Supplementary Table 2: the effect of variables with missing information (i.e., intervention phase and the location of primary tear) was assessed by conducting multiple imputation with 10 imputations performed.
