# Supplementary material for: Prognostic Value of the Systemic Immune Inflammation Index after Thoracic Endovascular Aortic Repair in Patients with Type B Aortic Dissection
Source: Dis Markers. 2023 Feb 17;2023:2126882. doi: 10.1155/2023/2126882 (PMC9957628; doi:10.1155/2023/2126882)
Supplement: Supplementary Materials — Supplementary Figure 1: the Kaplan–Meier reintervention-free survival curves after thoracic endovascular aortic repair (TEVAR) with the SII of >2893 versus <2893. Dashed lines indicate the upper and lower limits for 95% confidence interval (CI). Graphical Abstract/Supplementary Figure 2: although perioperative inflammation has a role in predicting postoperative outcomes after thoracic endovascular aortic repair (TEVAR) for type B aortic dissection, yet inflammatory biomarkers have not been incorporated in any risk stratification model. Therefore, a further study of postoperative inflammatory biomarkers is demanded. This study reveals that elevated postoperative systemic immune inflammation index (SII) and age are independent risk factors for aorta-related adverse events after TEVAR in type B aortic dissection. It is indicated that SII, an easily measured biomarker in clinical practice, has a certain boundary value, beyond which the risk of aorta-related adverse events approximately doubled. Supplementary Table 1: the study population was restricted to patients with acute aortic dissection to minimize the potential difference between acute and chronic dissection. Supplementary Table 2: the effect of variables with missing information (i.e., intervention phase and the location of primary tear) was assessed by conducting multiple imputation with 10 imputations performed. [file 2126882.f1.zip › Supplementary table (1).docx]

**Supplementary Table 1. Multivariable Cox regression: acute dissection**

|  | HR (95% CI) | P-value |
| --- | --- | --- |
| Age | - | 0.054 |
| NLR | - | 0.241 |
| MLR | - | 0.644 |
| PLR | - | 0.452 |
| **SII** | **1.914 (0.989-3.704)** | **0.054** |
| SIRI | - | 0.481 |

NLR = Neutrophil-to-lymphocyte ratio; MLR = Monocyte-to-lymphocyte ratio; PLR = Platelet-to-lymphocyte ratio; SII = Systemic immune inflammation index; SIRI = Systemic inflammatory response index; OR = odds ratio; CI= conﬁdence interval.

The study population was restricted to patients with acute aortic dissection to minimize the potential difference between acute and chronic dissection.

**Supplementary Table 2. Multivariable Cox regression: multiple imputation**

|  | HR (95% CI) | P-value |
| --- | --- | --- |
| Age | - | 0.211 |
| NLR | - | 0.175 |
| MLR | - | 0.262 |
| PLR | - | 0.113 |
| **SII** | **2.010 (1.443-3.174)** | **0.048** |
| SIRI | - | 0.384 |

NLR = Neutrophil-to-lymphocyte ratio; MLR = Monocyte-to-lymphocyte ratio; PLR = Platelet-to-lymphocyte ratio; SII = Systemic immune inflammation index; SIRI = Systemic inflammatory response index; OR = odds ratio; CI= conﬁdence interval.

The effect of variables with missing information (ie, intervention phase and the location of primary tear) was assessed by conducting multiple imputation with 10 imputations performed.
