# Supplementary figures and images for: Prognostic Value of the Systemic Immune Inflammation Index after Thoracic Endovascular Aortic Repair in Patients with Type B Aortic Dissection
Source: Dis Markers. 2023 Feb 17;2023:2126882. doi: 10.1155/2023/2126882 (PMC9957628; doi:10.1155/2023/2126882)

## Slide 1
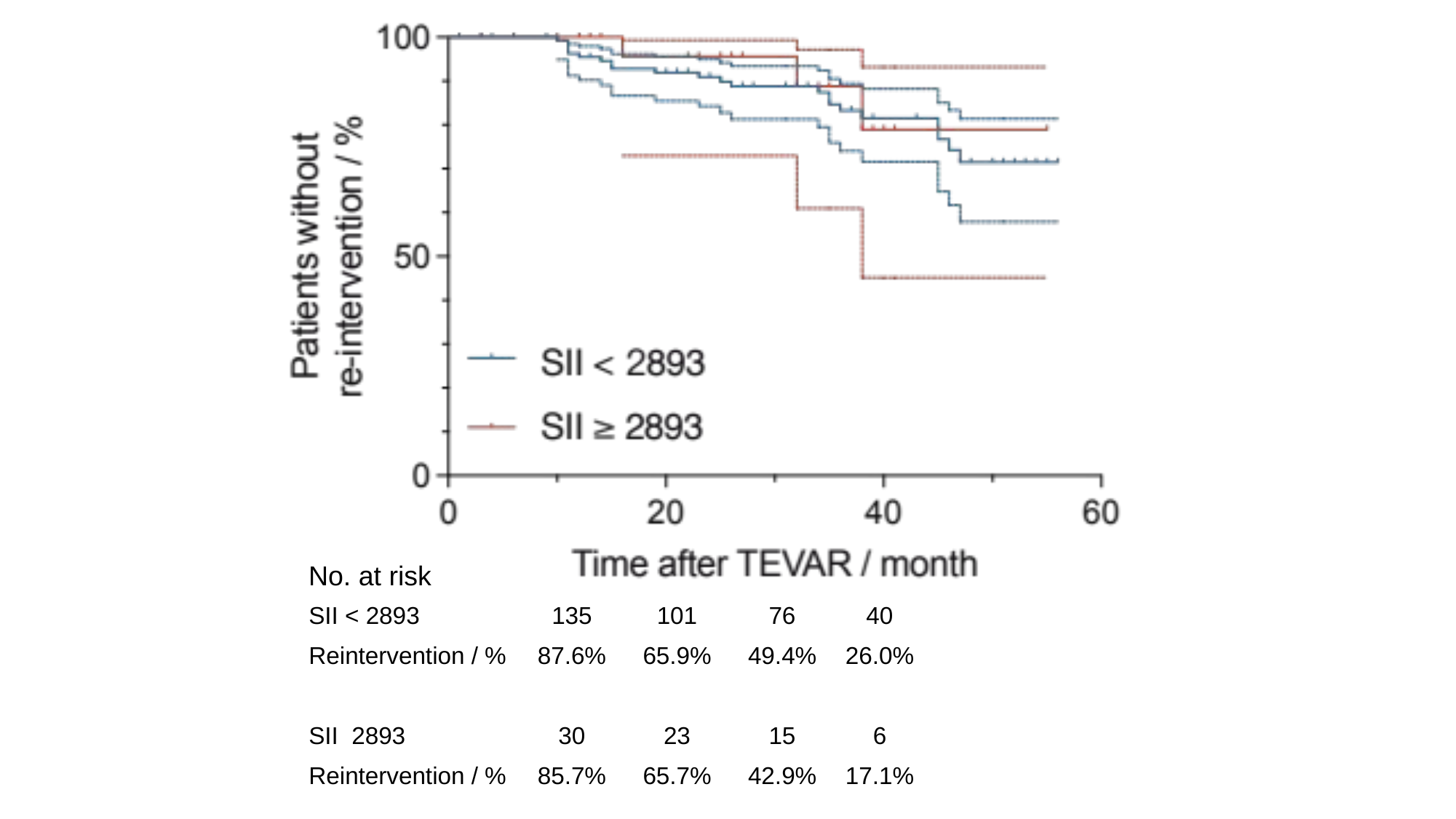

No. at risk

Supplement: Supplementary Materials — Supplementary Figure 1: the Kaplan–Meier reintervention-free survival curves after thoracic endovascular aortic repair (TEVAR) with the SII of >2893 versus <2893. Dashed lines indicate the upper and lower limits for 95% confidence interval (CI). Graphical Abstract/Supplementary Figure 2: although perioperative inflammation has a role in predicting postoperative outcomes after thoracic endovascular aortic repair (TEVAR) for type B aortic dissection, yet inflammatory biomarkers have not been incorporated in any risk stratification model. Therefore, a further study of postoperative inflammatory biomarkers is demanded. This study reveals that elevated postoperative systemic immune inflammation index (SII) and age are independent risk factors for aorta-related adverse events after TEVAR in type B aortic dissection. It is indicated that SII, an easily measured biomarker in clinical practice, has a certain boundary value, beyond which the risk of aorta-related adverse events approximately doubled. Supplementary Table 1: the study population was restricted to patients with acute aortic dissection to minimize the potential difference between acute and chronic dissection. Supplementary Table 2: the effect of variables with missing information (i.e., intervention phase and the location of primary tear) was assessed by conducting multiple imputation with 10 imputations performed. [file 2126882.f1.zip › Supplementary figure 1.pptx]
